# Large-scale research infrastructure projects: A conceptual review for science policy and management

**DOI:** 10.1177/00368504241266555

**Published:** 2024-12-04

**Authors:** David C Eggleton

**Affiliations:** Science Policy Research Unit (SPRU), 1948 University of Sussex, Brighton, UK

**Keywords:** Research infrastructure, theory, big science, megascience, large international science project, literature

## Abstract

This article reviews ‘large-scale research infrastructures’ work and other relevant literature from the science policy and management domains. Through a systematic literature review, the study identifies that there are no firm inclusion or exclusion criteria for a large-scale research infrastructure. The findings identify the need for filling this knowledge gap to support future analyses for large-scale research infrastructures to help scientists and science policymakers understand, plan, and evaluate their own work. A refined version of one of the concepts examined in this article, the ‘large international science project’, provides the most fruitful starting point.

## Introduction

This article uses a systematic literature review to examine how Large-Scale Research Infrastructures are characterized and defined in the scientific, science policy, and management contexts and to discuss some of the main issues that may determine their outcomes. Large Scale Research Infrastructures (LSRIs) are broadly conceived as a specific subtype of Research Infrastructure (RI), a resource intended to facilitate research and/or development work,^1^ of significant scale. Previous examples of LSRIs investigated in the literature include the Large Hadron Collider (LHC)^a^ at the European Organisation for Nuclear Research (CERN),^3^ the Institut Laue-Langevin,^b,^^4^ and the Swiss Initiative in Systems Biology^c^ (SystemsX.ch).^5^ The commonalities in these facilities in terms of their financial commitment over an extended timespan, the large workforces, and the intended use for research makes them an object of interest for the science policy community. From an academic perspective, the question over what exactly scale refers to is rarely made explicit in LSRI work with the definition of ‘large’ never fully explained.^4,6^

From a policymaking perspective, LSRIs consume a disproportionate amount of public funds relative to their small number and their outcome can have major implications for a national research system; the collapse of such a project can precipitate decline of entire research fields in a country as researchers cluster around the most advanced facilities in addition to the opportunity cost for the public investment.^7^ Both academic and policymaking documents acknowledge that projects of sufficient scale require different management approaches,^8^ yet in the case of LSRIs there are no clear guidance as to where this boundary lies. This creates a risk that inappropriate assumptions can be made as a result of incorrect categorization, risking project failure with all the consequences identified above; this occurred in the specific case of the Superconducting Super Collider (SSC) in the USA. This project was cancelled after costs had ballooned from an estimated US$6 billion in construction costs in the 1980s to over US$10 billion at the time of cancellation in 1993 and have been attributed to poor understanding in the management of a scientific project of this scale.^7^ This justifies a mapping the different conceptions of LSRIs to provide such categorizations. As this article demonstrates, other concepts can be used to operationalize ‘large’ and ‘significant scale’ in terms of big budgets, extensive staffing, significant infrastructure, and substantial laboratories.^9^ These operationalizations facilitate links between LSRIs and the project management domain as there is overlap between the budgetary requirement of at least one billion US dollars between one particularly useful concept examined for this article, the Large International Science Project (LISP),^10^ and the megaproject body of knowledge.^8^ The article also discusses practical science policy and management issues that can affect LSRIs with specific consideration given towards membership policy and cost distributions.

The RI domain has a very broad definition with scientific instruments, databases, and even library archives considered as RIs.^11,12^ RIs can be single-sited, distributed, or even virtual.^1^ Both LSRI and RI are terms that arose from policymaking rather than being science-led: the scientific terminology arose in response to these developments.^13^ Nonetheless the central issue exists that there are no firm inclusion criteria defining an LSRI with some literature using extremely vague definitions simply stating that an LSRI is large.^4,6^ This article seeks to begin the process of developing such inclusion criteria by mapping out existing concepts in the literature through answering the question – ‘*What criteria are used to conceptualize large scale research infrastructure?’*.

Although no consensus exists regarding the definition of LSRIs amongst scientists and policymakers, the key concepts that differentiate an LSRI from other RIs are clearly scale, both physical and investment, and technical complexity.^4^ Those LSRIs that have been examined in the literature are substantial facilities with construction budgets in the billion US dollar range and they are often organized as international collaborations due to the necessary investment being beyond the funding capacity of any single national research system.^14^ These LSRIs are directly relevant to policy interest as a vehicle for investing public funds into knowledge production, creating or sustaining innovation ecosystems, or societal change.^15,16^ However, there is a clear bias towards certain kinds of LSRIs as most apparatus identified in the preliminary reading and this literature review are predominantly big capital facilities in the physical sciences.^4,17^

The scale of resources and construction costs required to realize the experimental aims has often made it unrealistic for a single national budget to bear such expenditures in excess of one billion US dollars.^17,18^ Greater internationalization through various disparate legal approaches has been one response to this issue.^10,19^ The traditional legal form structures the laboratory as a corporation; alternative legal models do exist whether as an Intergovernmental Organization, wherein national governments agree treaties or conventions to create an autonomous research-dedicated organization,^20^ or as a European Research Infrastructure Consortium (ERIC) – which provides a standardized legal and organizational structure that can be used by any RI.^21^

Technical complexity and high technological uncertainty are LSRI characteristics that make them relatively novel compared to other large projects. Many RIs incorporate previous generations of machines into the supporting infrastructure of the current generation.^22^ LSRIs share this characteristic but at a much larger scale. Issues around complexity in LSRIs have been examined by Kaufmann et al.^5^ and in the context of a major scientific laboratory by Whyte et al.^23^

LSRIs are also socially complex with global collaborations and communities linked to them; Traweek^24^ and Zabusky^25^ conducted notable ethnographies into high energy physicists and space scientists respectively. However, it must be noted that in both cases the unit of analysis was the laboratory rather than the apparatus which is the primary focus of this article. Of equal interest for understanding the social construction of experimental collaborations is Boisot et al.^26^ where the experiment is the unit of analysis. Other work has examined the social composition of laboratories from the perspective of the history of science,^7,27,28^ or by examining the role played by leading figures in shaping their institutions and experiments.^29,30^

Many studies of LSRIs examine their scale and significant potential for spillover effects into the wider economy.^31^ Several different terms have been used to refer to them, but in every case, the lack of established conceptual frameworks for examination of LSRIs still creates empirical inclusion challenges.

Despite LSRIs being relatively few, they consume a disproportionate amount of funding compared to relatively smaller RIs.^32^ Therefore, understanding their dynamics including from project management, research evaluation, and organisational studies perspectives may help enhance their efficiency and impacts. As this systematic literature review shows, there are no firmly agreed criteria defining an LSRI with some literature using a definition that simply says LSRIs are ‘large’ with no further clarifications offered.^4,6^ This is insufficient for research and policymaking purposes which justifies this article seeking to systematically review and map the relevant concepts to provide a starting point for the development of such criteria. This article examines this gap and proposes that the large international science project (LISP) concept is the most appropriate starting point for future conceptualizations. This acts as a beginning for future science policy research to support scientists and policymakers in their work.

The remaining sections of this literature review are structured as follows: Section 2 summarizes the methods used to obtain the literature sample that informed this review. Section 3 provides an overview of existing conceptions of LSRIs taking a broad approach of the literature; Section 4 discusses the findings and examines other LSRI issues that are relevant to science policy and management; and Section 5 concludes the review.

## Review: Methods and data

This section presents the methodology including the sampling strategy and inclusion criteria by which an article was included in the systematic literature review. This section concludes with some descriptive statistics concerning the literature sample including the key journals and chronology of the articles.

### Research objectives

This systematic literature review sought to identify work that examined LSRIs whether from a management or science policy perspective to determine the inclusion and exclusion criteria. However, it was acknowledged early on that scientists themselves may write such accounts based on personal experiences so the systematic literature review took a broad approach towards data collection to avoid excluding potentially valuable insights from such practitioners.

### Sampling strategy and inclusion criteria

The article for this research adopted a systematic review approach, this ensures that the selection and study review phases of this research can be replicated.^33^ The initial sample was obtained by incorporating the terms shown in Table 1 as a Web of Science (WoS) Core Collections query. This query returned an initial sample of 1326 English language results. This sample was downloaded with the abstract and keywords manually reviewed for final inclusion in the study. An article was included in the literature review if it met one or more of the following criteria: (1) the article focus was clearly on one or more LSRIs; (2) the topic examined issues that would be valuable for clarifying any allied issues; or (3) if the same term was used to describe different phenomena to help understand the effect of disciplinary context on big science as a terminology. This process led to a core sample of 205 articles that contributed to this review.

**Table 1.** Search terms used to collect the initial literature sample.

Terms
“large scale research infrastructure”
“large international science projects”
“megascience”
“big science”

One issue that emerged during the reviewing was the dual use of ‘big science’ as referring to both large scale scientific research and to a separate phenomenon characterized by the exponential growth in scientific knowledge generation which should be differentiated.^34^ This latter model of scientific growth was further refined and is regarded as one of the founding works in the quantitative studies of science or scientometrics.^35^ This review avoided this issue by using the definition of big science to refer to the first concept which requires at least one and usually most of the following: big budgets, extensive staffing, significant infrastructure, and substantial laboratories.^9^

### Data synthesis and analysis

**Figure 1.**
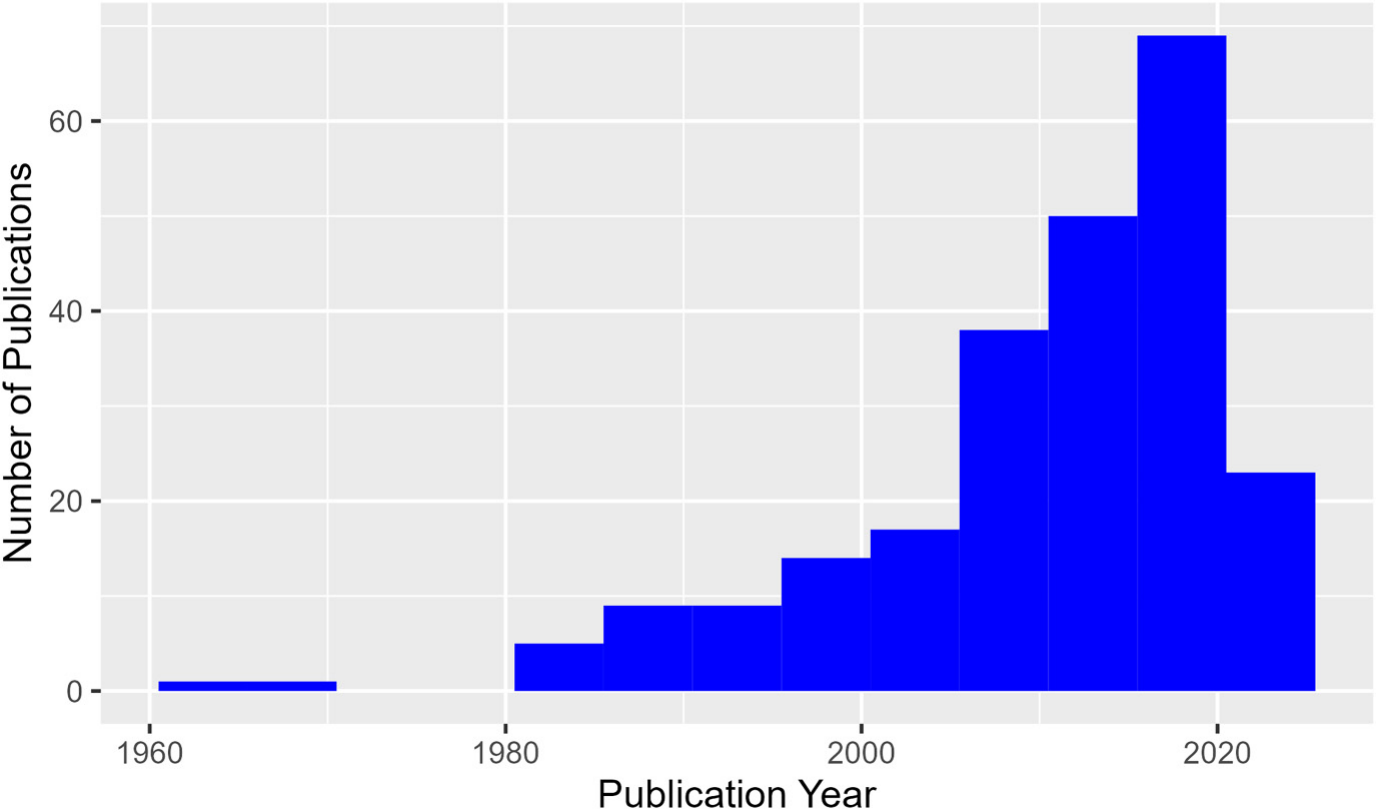
Studies included in the sample by year (N = 205).

Interest in large-scale research infrastructure and its associated terms has increased significantly over the last 40 years or so as can be seen in Figure 1 which shows graphically the publication year of articles in the sample. These articles are widely distributed with over half of the articles coming from journals with a single relevant article (Table 3).

Equally as can be seen below in Table 2, almost 75% of the included studies were completed in the last 20 years and over 90% since 1992^d^ when the OECD established the ‘Mega-science Forum’^e^ to facilitate international cooperation in large international science projects.^36^ During the systematic literature review it became a concern that this Mega-science Forum may result in a pool of articles that examined the forum rather than the apparatus but this proved to be unfounded. It appears that the increasing level of interest in the topic is likely a result of increasing government scrutiny towards major science investments after the 1993 failure of the SSC at the cost of US$10 billion^7^ This failure provided evidence that large-scale research infrastructure had become too big and complex for any single national government.

**Table 2.** Number of studies organised according to publication year.

Publication Year	Number of Studies	Percentage	Cumulative Percentage
1961	1	0.5	0.5
1968	1	0.5	1.0
1983	1	0.5	1.5
1985	4	2.0	3.5
1986	2	1.0	4.5
1987	2	1.0	5.5
1988	3	1.5	7.0
1989	1	0.5	7.5
1990	1	0.5	8.0
1991	1	0.5	8.5
1992	2	1.0	9.5
1993	1	0.5	10.0
1994	3	1.5	11.4
1995	2	1.0	12.4
1996	1	0.5	12.9
1997	2	1.0	13.9
1998	4	2.0	15.9
1999	3	1.5	17.4
2000	3	1.5	18.9
2001	2	1.0	19.9
2002	2	1.0	20.9
2003	4	2.0	22.9
2004	5	2.5	25.4
2005	2	1.0	26.4
2006	9	4.5	30.8
2007	2	1.0	31.8
2008	6	3.0	34.8
2009	8	4.0	38.8
2010	8	4.0	42.8
2011	5	2.5	45.3
2012	5	2.5	47.8
2013	6	3.0	50.7
2014	10	5.0	55.7
2015	10	5.0	60.7
2016	15	7.5	68.2
2017	12	6.0	74.1
2018	8	4.0	78.1
2019	13	6.5	84.6
2020	10	5.0	89.6
2021	12	6.0	95.5
2022	6	3.0	98.5
2023	3	1.5	100.0

**Table 3.** Number of studies included in the systematic literature review by journal (N = 205**)**.

Publication Title	Number of Studies	Percentage (%)
SCIENTOMETRICS	10	4.9
RESEARCH POLICY	7	3.4
SCIENCE	7	3.4
PHYSICS IN PERSPECTIVE	6	2.9
HISTORICAL STUDIES IN THE PHYSICAL AND BIOLOGICAL SCIENCES	5	2.4
NATURE	5	2.4
SCIENCE AND PUBLIC POLICY	5	2.4
GREEN PROCESSING AND SYNTHESIS	4	2
MINERVA	4	2
PHYSICS TODAY	4	2
SOCIAL STUDIES OF SCIENCE	4	2
TECHNOVATION	4	2
CURRENT SCIENCE	3	1.5
FUSION ENGINEERING AND DESIGN	3	1.5
JOURNAL OF TECHNOLOGY TRANSFER	3	1.5
ACTA PHYSICA POLONICA B	2	1
EMBO REPORTS	2	1
HISTORY OF THE HUMAN SCIENCES	2	1
INDUSTRIAL AND CORPORATE CHANGE	2	1
IUCRJ	2	1
JOURNAL OF INFORMETRICS	2	1
JOURNAL OF LIBRARIANSHIP AND INFORMATION SCIENCE	2	1
PHYSICS WORLD	2	1
PLOS ONE	2	1
PUBLIC UNDERSTANDING OF SCIENCE	2	1
REVIEW OF POLICY RESEARCH	2	1
SCIENCE AS CULTURE	2	1
SCIENCE TECHNOLOGY & HUMAN VALUES	2	1
STUDIES IN HIGHER EDUCATION	2	1
Other (Fewer than 2 articles)	103	50.2
Total	205	100

## An overview of existing LSRI conceptualizations

This section examines the most relevant literature examining LSRIs. The primary concepts that inform this literature review are the Large International Science Project (LISPs), ‘Big Science’ projects, Megascience, and the LSRI concepts. While there are other potential conceptions of LSRIs such as Major Research Equipment and Facilities Construction (MREFCs) or ESFRI landmarks, the literature searched revealed that these statuses are primarily intended to allow RIs to benefit from specific funding accounts or to showcase key offerings and there is no academic literature to review. Most accounts written by scientists take the form of personal recollections or function as technical updates.^39,40^ Others seek to develop a roadmap to outline future facilities, while others draw on past experiences to identify project management improvements.^42^

### Large International Science Projects (LISPs)

The Large International Science Project (LISP) concept will be familiar to some policymakers but has fallen into relative disuse.^19^ Its defining characteristics are that at least two countries must agree to collaborate together and that projects should exceed $US1 billion in construction costs.^10^ Previously, the LISP concept has been used to examine technological uncertainty,^43^ cultural challenges,^44^ maintaining funding,^45^ and membership policy.^10,46^ However, the requirement for international collaborations means that large scientific projects that are funded solely by a domestic funder are outside its scope. Given the significant funds still invested by single countries into research infrastructure this somewhat limits its utility as a concept. However, the clear budgetary criteria and its preexisting links with technological uncertainty make a revised version of this concept valuable for conceptualizing the LSRI domain.

### Big science

The term ‘Big Science’ for this article characterizes an organizational change where entire laboratories with workforces numbered in hundreds or thousands became devoted to a single research agenda although no formal budget limit exists.^47^ The emphasis in this context is on large pieces of capital equipment including particle accelerators or nuclear reactors and tends to result in only physical science apparatus in the latter half of the twentieth century being considered ‘big science’. A comparatively recent development over the past 20 years has been that life sciences facilities have being described as big science.^48^ Big science has also been the term used for analyzing scientific collaborations,^49^ for understanding laboratory-industry partnerships,^50^ and for understanding path trajectories of big science.^51^

This term ‘big science’ has become extremely pervasive both in research and in the public imagination.^52^ These major investments became the subject of research evaluation exercises to understand the return on these investments.^53^ Such evaluations have examined the returns from scientometric perspectives,^54,55^ economic perspectives,^56,57^ and more recently as tools for tackling major societal challenges.^58,59^

### Megascience

Despite the popular reference to the growth in scientific outputs and laboratory size as ‘Big Science’ noted above,^34,47^ some historians of science distinguish between ‘Big Science’ and ‘Megascience’.^27^ Such historians argue that megascience evolved out of big science in response to aggressive government budget constraints in the 1970s.^27^ Larger projects, notably those at the American particle physics laboratory Fermilab in the 1970s, opened up new avenues of scientific enquiry to secure long-term government funding.^27^ The increase in size and scope of these particle physics projects and experiments soon made it difficult to identify clear project endpoints.^27^ Each experiment led to a need for further upgrades to answer additional questions, so judging the end of an experiment became problematic. Other work characterizes megascience as the effect of megatrends affecting scientists and their work.^60^ However, no firm inclusion or exclusion criteria exist that can be readily applied by science policy researchers.

In the policy domain, the term ‘megascience’ first emerged as the name for the Organization for Economic Cooperation and Development's (OECD) science working group. This ‘Mega-science Forum’ focused on scrutinizing science policy issues and catalyzing international scientific collaboration with a particular emphasis on the physical sciences.^36^ The term has been used in technical works,^61^ in social science,^62^ and to gain a reader's interest in general science work.^63^

### Large Scale Research Infrastructures (LSRIs)

The domain of LSRIs shares some overlap with that of ‘Big Science’ projects examined above. However, Big Science tends to focus on the physical sciences or to the rapid generation of scientific knowledge in the post-war period depending on the context^34,47^; whereas LSRIs can be linked to a far broader range of disciplines.^4,5^ Yet from the literature sample it is notable that these broader disciplines are limited to systems biology,^64^ neuroscience and IT,^46^ and environmental research^49^; all of which are life sciences-related. The issue over why LSRIs can be linked to essentially any research field yet the literature sample was composed of relatively few research fields is considered in the discussion section. The defining characteristics of LSRIs are described in broad terms as linked to their scale, wider impact, and collaborative character.^3,4^ However, the formal inclusion criteria is extremely vague. Although Qiao et al.^6^ proposed a definition of LSRIs which used by D’Ippolito and Ruling,^4^ the precise inclusion criteria were unclear as the definition was diluted down to something that is ‘large’. Equally Kaufmann et al.^5^ investigated the failure to construct a LSRI but did not seek to define formally the boundaries for the category.

The most common type of literature from the sample using LSRI terminology is technical updates written by scientists.^40^ Some workstreams that inform LSRI issues include strategies for understanding and designing LSRIs,^5^ studies examining the social structure of such collaborations,^4^ and the returns on LSRI investments to the wider economy and society.^16^

### Summary of the main findings

The systematic review of existing literature on LSRIs is summarized in Table 4. This facilitates understanding that existing work relevant to LSRIs broadly defines the concept with only a single concept providing robust inclusion criteria. What can be said is that the criteria used for delineating the two types of big science are highly salient to conceptualizing LSRIs.^9^

**Table 4.** Summary of the inclusion criteria identified from the literature review.

Concept	Criteria
Large International Science Projects	Minimum budget of $1 billion US dollars, two or more countries must collaborate together, significant technological uncertainty
Big Science	Laboratories with large workforces devoted to a single research agenda, traditionally physical sciences-oriented but increasingly including biological sciences as well
Megascience	No clear project or experimental endpoint, predominantly physical sciences-oriented
Large-Scale Research Infrastructure	Facilities or instruments that are ‘large’, traditionally linked to big science but no disciplinary restrictions

Based on the above, LSRIs can be defined in terms of scale both in terms of capital investment, workforce size, and the lifetime of the facility. There is also a disciplinary component with most literature examining LSRIs examining physical sciences facilities although biological sciences have emerged as an area of interest. This is likely to be the product of the few LSRIs happening to be in the physical sciences rather than because of deliberate exclusion. But these criteria are generally quite vague with only the ‘LISP’ concept devising any kind of formal inclusion criteria.

## Discussion

This section draws on the findings examined above to discuss the findings and to examine other practical issues that may impact LSRI. It also examines what other literature domains could be drawn on to devise more formal criteria for what may be considered an LSRI.

This review has identified that issues around a lack of inclusion criteria around what may be considered an LSRI are common across multiple conceptualizations of such facilities. While the commonalities in terms of big apparatus, big workforces, and big budgets are definitely present, the individual researcher is primarily left to devise the inclusion criteria by themselves. Other literature using certain concepts, notably big science and megascience, almost exclusively use such concepts to refer to physical sciences with life sciences present in the literature as noted above but as a secondary topic.

A further issue identified during this review has been that while LSRIs can be linked to essentially any research field, the literature sample examining this topic specifically contained material almost exclusively related to physical and life sciences. Why this might be is not entirely clear but a few possibilities exist. One reason could be simply because of the cost of doing research with physical and life sciences simply requiring much larger research infrastructures to answer their research questions. A second possibility could be as a result of science policy decision making. Governments have tended to emphasize life sciences research on the grounds of improving quality of life for citizens; for example the ‘war on cancer’ in the US having been ongoing since the 1970s under a series of terminologies with annual spending on cancer research and development estimated to be US$307 billion by 2026.^65^ It would therefore not be surprising for life sciences to have developed extremely expensive research infrastructure to leverage these funds. A third possibility could be because the LSRIs considered in this article are predominantly in the public sector; it is therefore perfectly possible that there are hidden private research infrastructures that are not captured through this literature review. Future research could examine this issue to obtain clarity.

The LSRI field is an area where examining the relevant general project management literature around complexity and megaprojects may become relevant. The LISP concept in particular shares the same budget criterion as megaprojects which is a minimum budget size of one billion US dollars.

This LISP concept is however extremely powerful given its firm requirements for a budget of at least one billion US dollars and the link with project and technical complexity. Such requirements serve to link LSRIs to a much broader range of literature in the management domain; in particular the budget requirement and a significant level of technological uncertainty maps onto the megaproject and technological complexity bodies of knowledge respectively.^8,66^

There are several practical challenges to LSRI construction and operation of direct relevance to a science policy and management community. Firstly, in terms of membership policy. The notion that an LSRI may have members from all or most countries on a continent is a very rare one with few notable exceptions. A more frequent tendency, particularly in Europe, is for smaller consortia formed of approximately twelve countries.^37^ Current research indicates that there are four variables affecting decisions for national governments; These are economic, scientific, cultural, and political factors.^10^ In terms of economic forces, Vincenzi and Shore^10^ proposed that LSRI membership is negatively correlated with government bond interest rates. On a more practical level a powerful economic consideration for joining an LSRI is that in many cases only corporations based in member states are eligible for supply contracts,^67^ with potential positive feedback loops in terms of creating and sustaining a high technology industry. In scientific terms, potential member states evaluate the trade-offs and potential opportunity costs linked to LSRI membership. While there are obvious risk and cost sharing efficiencies linked to LSRIs, discoveries from international collaborations dilute any national prestige compared to an independent effort.^10^ Cultural factors examine how ‘forward looking’ the civil society is with greater levels of future orientation associated with greater propensity to stay in international collaborations for the long term. Political factors refer to the level of high level political support given towards international collaboration,^10^ with considerations towards whether these scientific collaborations could be leveraged to achieve foreign policy goals on a ‘science for diplomacy’ basis.^68^

A second issue affecting LSRI management and science policy relates to cost distribution, traditional methods for their allocation are based on GDP, by utility to the national scientific community, or on the likely benefits to domestic industry on a ‘fair return’ principle.^69^ However, an ongoing tension on this issue is that the host member states seem to benefit disproportionately as a result of having the laboratory on their territory – such benefits are not captured by more traditional cost distribution mechanisms with novel ‘host state contributions’ developed to mitigate.^69^ Other funding sources can be accessed through the creation of associate or observer membership status when full member status is unlikely to be acceptable to trade cash for limited scientific access.^69^ The composition of LSRI funding can also be a challenge; from a research institutions perspective it is more preferable for members to make ‘cash’ contributions on the grounds of flexibility, it is more common that a member state contributes on an ‘in-kind’ basis. Such in-kind contributions are often accompanied by a provision that the funds must be spent in that member state with reporting requirements to verify the provisions have been adhered to.^49^

This article makes contributions to LSRI theory by examining the most relevant concepts to provide a starting point for inclusion criteria for future work. From the above, it is apparent that the LISP concept has the clearest criteria to link future work to with the project management literature given the clear conceptual link with the megaproject domain.^8^ Curren research has examined these concepts in isolation and this article delivers a clarification of their interrelatedness. While scale and complexity are the classic hallmarks of an LSRI, the LISP concept has the most robust set of criteria linking the LSRI domain to the broader project management literature. However, the internationalized requirement may be limiting by excluding LSRIs funded by a single government such as in the USA or China.^6^ But future research could adopt aspects of the LISP concept while overlooking this international collaboration requirement.

An obvious limitation of this review is conceptual as LSRI projects are large in scale but few in number. It can therefore be easily argued that every LSRI is unique with no utility in understanding or developing inclusion criteria for future researchers. However, the size of these projects brings disproportionate impact on government budgets relative to their numbers. The success or failure of an LSRI can impact an entire research system – as an example some authors have claimed that the failure of the Superconducting Super Collider shifted high energy physics leadership from the USA to Europe.^7^ Equally, the majority of projects that would meet these criteria examined above tend to be located in western countries.

Future research could also formally categorize these LSRIs as a novel subcategory of megaprojects, which have a minimum budget requirement of at least one billion US dollars,^8^ that have the unusual additional characteristic of a ‘high’ or ‘super high’ level of technological uncertainty.^66^ These characteristics could result in LSRIs displaying exaggerated characteristics that are apparent in other megaproject. There is already some research that has looked at ‘science megaprojects’ from a science diplomacy perspective,^20^ and this represents an opportunity for fruitful developments.

A second potentially useful concept for future work is ‘Large Technical Systems’ (LTS).^70^ These large technical systems are conceptualized as major artefacts constructed by social-technical interactions to shape wider society.^70^ Physical or non-physical components interact with one another with these interactions having cascading consequences across the system.^70^ These systems are limited by controls either from physical artefacts or human operators.^70^ Future work analyzing LSRIs from a large technical systems approach represents an opportunity.

## Conclusion

The challenge that motivated this article relates to conceptualization and therefore inclusion criteria of LSRIs – namely, what is the lower dividing point at which a research infrastructure is considered to have become ‘large’? Although some definitions have been proposed and used in science policy research, there were no clear inclusion criteria. Equally, many other LSRI studies have been conducted which did not even consider the issue of inclusion criteria. The individual researcher therefore lacked clarity regarding the definition and extent of ‘large’ in this context. Through a systematic literature review, this work has examined the various concepts and identified that the LISP concept allows LSRIs to be mapped into other conceptual frameworks in the management literature and examined the practical challenges associated with LSRI construction, primarily in terms of membership policy and funding allocations.

## Abbreviations

CERN: European Organisation for Nuclear Research
ERIC: European Research Infrastructure Consortium
ICFA: International Committee for Future Accelerators
ISC: International Science Council
LHC: Large Hadron Collider
LISP: Large International Science Project
LSRI: Large-Scale Research Infrastructure
LTS: Large Technical System
MREFC: Major Research Equipment and Facilities Construction
OECD: Organization for Economic Cooperation and Development
RI: Research Infrastructure
SSC: Superconducting Super Collider
WOS: Web of Science

## Notes

a. The LHC is a collider machine which is used by High Energy Physicists to understand the composition of subatomic particles and provide experimental evidence to determine the validity of theoretical predictions. At the time of its commissioning, its construction cost was estimated to be 6.5 billion Swiss Francs.^2^

b. The Institut Laue-Langevin is a physics facility used for neutron diffraction and neutron scattering with an annual budget of €100 million and an overall investment in the €2 billion range.^4^

c. SystemsX.ch was a major research initiative by the Swiss government to provide a common data repository for systems biologists with a program spend of 220 million Swiss Francs over its twelve year lifespan.^5^

d. Where such publication year data was present. It is acknowledged that four of the included 205 studies did not contain this data.

e. Now known as the OECD Global Science Forum.
